# Evaluation of the Scored Questionnaire to Identify Individuals with Chronic Kidney Disease in a Community-based Screening Program in Rural North Carolina

**DOI:** 10.4172/2161-0711.S2-007

**Published:** 2014-03-28

**Authors:** Donna H Harward, Heejung Bang, Yichun Hu, Andrew S Bomback, Abhijit V Kshirsagar

**Affiliations:** 1 Director of Education and Outreach, University of North Carolina at Chapel Hill, USA; 2 Professor, Clinical trials and observational studies, University of California Davis, USA; 3 Assistant Professor of Clinical Medicine, Columbia University, USA

**Keywords:** Chronic kidney disease (CKD) risk assessment, Community-based CKD screening, Chronic kidney disease risk self-assessment

## Abstract

**Background:**

Just over 10 percent of US adults over twenty years of age have chronic kidney disease (CKD). Early detection is essential to delay or halt CKD's progression, but screening and early detection of CKD in high risk populations is inconsistent, especially in rural and underserved communities.

**Objective:**

The objective of this study was to evaluate the effectiveness of the Screening for Occult Renal Disease questionnaire as a simple, self-report tool to identify individuals with increased likelihood of prevalent CKD in a rural North Carolina setting.

**Methods:**

Over an eight month period, in the context of the Kidney Education Outreach Program (KEOP), sixteen CKD screenings were conducted in two underserved, rural NC communities. For this study, the SCORED questionnaire was administered prior to the execution of the regular KEOP screening protocol.

**Results:**

For 172 participants for whom both blood and urine specimens were collected, there were fifteen (8.7%) who demonstrated less than normal kidney function. The SCORED sensitivity and specificity were 100% and 42%, respectively. The positive predictive value was 14% and the negative predictive value was 100%. The positive likelihood ratio for low eGFR was 1.7 and conversely, the negative likelihood ratio for low eGFR was zero.

**Conclusion:**

In this study, the SCORED performed comparably to previous settings in established datasets and cohort studies, with high sensitivity and negative predictive values that allow for ruling out the presence of disease. SCORED appears to provides a practical alternative to the administration of regular CKD screening protocols that can be difficult to organize and administer in rural settings. The need for further evaluation of SCORED in underserved, high-risk communities is recommended.

## Introduction

Chronic kidney disease (CKD) affects 10 per cent of the American population and persons of African American, Hispanic or Asian ancestry are at increased risk for developing CKD [1]. The international estimated CKD prevalence rate of 8-16% establishes CKD as a worldwide public health concern and further warrants the development of effective, self-administered interventions to identify early-stage chronic kidney disease among at-risk populations [2]. Even though diabetes, hypertension, heart disease and a family history of kidney disease are the primary risk factors for developing CKD, awareness about the disease remains low among primary care providers and at-risk persons [3-5]. Low awareness results in delayed intervention by primary care providers and decreases the likelihood of patients' self-management of CKD's associated chronic illnesses [6,7]. CKD screening is promoted as an important aspect of CKD educational intervention and the United States Preventive Services Task Force recommends screening for high-risk populations [8,9].

North Carolina (NC) consistently has one of the ten highest end-stage kidney disease (ESKD) prevalence rates among the fifty United States and the District of Columbia and rural, underserved communities register NC's highest ESKD rates [10]. Providing CKD education and screening to these at-risk populations is challenging: the percentage of uninsured persons is high; primary care providers are few; and, providing targeted populations with early intervention and targeted, community-based education involves multiple events [11,12]. CKD screening relies on semi-invasive measurements and is relatively expensive. This study's objective is to evaluate the SCORED questionnaire as a non-invasive, simple, self-report screening intervention among a rural at-risk population.

## Materials and Methods

**Figure 1:**
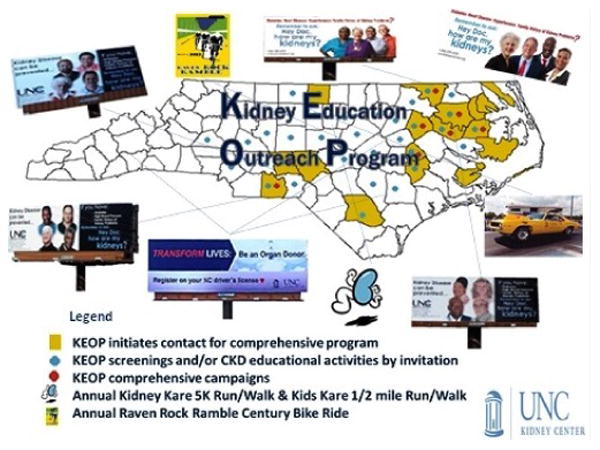
KEOP outreach in North Carolina.

**Figure 2:**
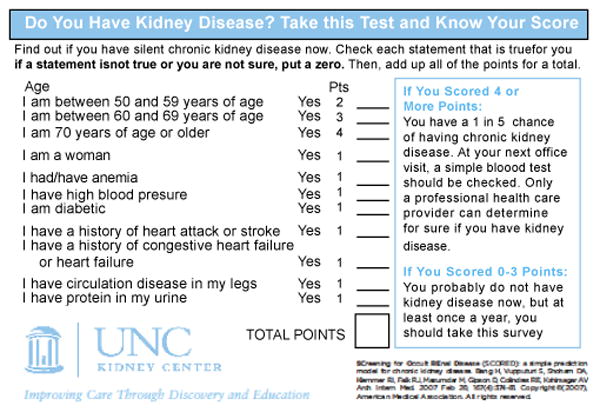
Screening for Occult REnal Disease (SCORED) Survey.

Study Design: This study was conducted in the context of an established, community-based CKD awareness and prevention program, the Kidney Education Outreach Program (KEOP) for this cross-sectional study (Figure 1). The SCORED questionnaire was completed by each study participant prior to the standard KEOP screening intervention regular KEOP screening protocol that includes focused medical history, urinalysis, venipuncture, and a personal consult (Figure 2). Each screening was hosted by a community partner (e.g., church, health department, primary care provider, civic group) [13,14].

Study period and setting: From December 2010 through August 2011, sixteen KEOP screenings were conducted across two rural, economically challenged counties with large minority populations.

**Figure 3:**
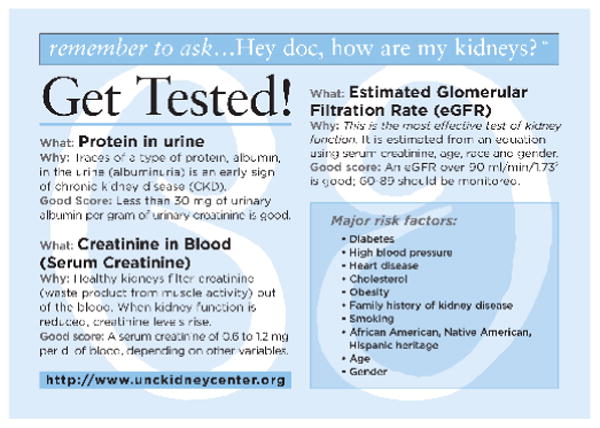
SCORED Educational Resource Card.

The SCORED has demonstrated test characteristics suitable for screening, with high sensitivity and high negative predictive value in retrospective studies using independent national and international populations [15,16]. SCORED asks for self-reported information on nine variables and assigns an integer value for each variable present (Figure 2). The reverse side of the laminated self-assessment instrument describes the three tests most frequently used to assess kidney function, explains their respective scores, and lists the major risk factors for developing CKD (Figure 3). Individuals from general healthy adult populations with a cumulative score ≥ 4 have demonstrated an approximately 20% chance of having CKD, defined as an estimated glomerular filtration rate (eGFR) of <60 ml/min/1.73^2^. However, SCORED has never been administered in a rural setting or within the context of an established, on-going community-based CKD health education intervention.

Urine was tested for albuminuria using the Roche® Micro albumin dip stick; proteinuria and glucose were assessed using Chem-strip 10 dip sticks and the Roche Urisys^®^ machine. Serum creatinine concentration was measured with the ISTAT^®^ point of care analyzer. Estimated GFR was calculated using the abbreviated Modification of Diet in Renal Disease formula [16]. The presence of ≥ 20 mg/l albuminuria was considered positive for proteinuria for the SCORED questionnaire if participants were unable to give a history of the condition. A total score of >4 was considered positive [17]. The sensitivity, specificity, positive predictive value (PPV), negative predictive value (NPV), positive likelihood ratio (LR+), and negative likelihood ratio (LR-) of the SCORED algorithm for participants with less than normal kidney function (eGFR<60 ml/min/1.73^2^) were calculated [18]. All analyses were performed using survey procedures in SAS statistical software, version 9.1 (SAS Institute Inc., Cary, NC).

## Results

Sixteen separate screening events were conducted over the seven month period of the study. The number of participants at each event ranged from 16-37 individuals, with the total number of screened participants being 360. Two hundred fifteen of these individuals tested positive or proteinuria. Blood was obtained for 172 of the 215 participants. Table 1 shows demographic characteristics for the entire screened cohort and the individuals with proteinuria. The two groups did not differ significantly by age, gender or race. The prevalence of eGFR<60 ml/min/m2 was 8 7% (15/172) of the participants tested. The sensitivity and specificity (Table 2) of the SCORED survey instrument were 100% and 42%, respectively. The Positive predictive value for a score >4 was 14% and the Negative predictive value for a score <4 was 100%. The SCORED sensitivity and specificity were 100% and 42%, respectively. The positive likelihood ratio for low eGFR with a questionnaire point score>4 was 1.7 and conversely, the negative likelihood ratio for low eGFR with a questionnaire point score of <4 was zero.

## Discussion

In this screening program in rural North Carolina, the SCORED algorithm performed comparably to previous settings in established national datasets and cohort studies. With its high sensitivity and negative predictive value, and low negative likelihood ratio, SCORED is well suited for ruling out the presence of disease. Researchers have previously relied on laboratory-based measures for kidney disease screening, e.g., urine protein or serum creatinine. For targeted, community-based screening in rural settings, urine and blood measurements may be less feasible due to limited resources and the need for multiple, staged events to reach the targeted population. Additionally, SCORED eliminates some of the challenges associated with collecting urine and blood specimens from persons who are unable to generate one or both specimens in a single field setting. In this study, twenty percent of the persons who tested positive for proteinuria either declined venipuncture or due to obesity, rolling or collapsed veins, venipuncture was not successful. A questionnaire such as SCORED appears to be a reasonable alternative to onsite, standard kidney function screening with the additional health education benefit of raising awareness about CKD and its associated risk factors. SCORED allows for easy and inexpensive dissemination to at-risk persons unavailable for an on-site kidney screening.

This study has limitations. First, the SCORED uses self-report medical histories that could not be confirmed. In practice, though, self-report has been adopted as the most common and efficient method of screening in community settings. Second, we could only use a single measurement of serum creatinine to assess estimated GFR. A single measurement likely over estimates the number of CKD cases, but also provides a conservative approach that may be ideal for CKD prevention purposes. Nonetheless, the prevalence of eGFR<60 ml/min/1.73 m^2^, 8.7% of those tested, was similar to national estimates of stages 3-5 chronic kidney disease. Lastly, we had a relatively small sample of study participants. Thus, while the performance characteristics are comparable to those sustained from large databases, a large study may be warranted, especially in populations with varying prevalence of chronic disease.

Nonetheless, the SCORED appears to provide a simple, easily disseminated screening tool for identifying persons with undiagnosed CKD in at-risk populations in rural, resource limited settings.

## Figures and Tables

**Table 1:** Baseline characteristics of the study population.

Characteristic	All Participants N=360	Positive for Microalbuminuria N=215
Mean Age (SD)	55 (14.7)	54.9 (14.7)
Female (%)	73%	72%
African American	82.1%	82.2%

**Table 2:** Disease Status-categorizes individuals by eGFR and the risk status from the SCORED algorithm (eGFR MDRD formula^a^).

Test Result	Present	Absent	Total
	eGFR<60	eGFR ≥ 60	
Positive (points ≥ 4)	15	90	105
Negative (points<4)	0	67	67
	15	157	172

a Estimated Glomerular Filtration Rate-Modification of Diet in Renal Disease formula
